# Intravenous Thrombolysis for Central Retinal Artery Occlusion: A Look at the Literature for the Emergency Medicine Physician

**DOI:** 10.7759/cureus.41878

**Published:** 2023-07-14

**Authors:** Zachary Webb

**Affiliations:** 1 Emergency Medicine, Huntington Hospital, Northwell Health, Huntington, USA

**Keywords:** intravenous thrombolytic therapy, ophthalmologic emergency, evidence-based medicine (ebm), ischemic stroke, emergency medicine, intravenous thrombolysis, central retinal artery occlusion (crao)

## Abstract

Central retinal artery occlusion (CRAO) is a subtype of ischemic stroke and true ocular emergency presenting with acute, painless, monocular vision loss. Typical findings include poor visual acuity (VA), impaired color vision, relative afferent pupillary defect, and on fundoscopic evaluation, retinal edema, cherry red spot, and occasionally visualization of retinal artery emboli. While there are no proven treatments for CRAO, options include orbital massage, hyperbaric oxygen therapy, and intra-arterial or intravenous thrombolysis (IVT). This study reviews the current literature on the efficacy of IVT for patients affected by acute, symptomatic CRAO and provides an up-to-date, evidence-based background for emergency physicians (EPs) who evaluate and manage these patients.

## Introduction and background

Central retinal artery occlusion (CRAO) is a subtype of ischemic stroke and a true ocular emergency that can lead to permanent vision loss. It was first described in 1859 by von Graefe, a Prussian ophthalmologist and founder of modern ophthalmology [1]. CRAO is rare, with an incidence of one to two cases per 100,000 [2,3]. It typically presents with painless, sudden, monocular vision loss [4]. Large artery occlusive disease, arterial emboli, hypercoagulable disorders, and calcific emboli are implicated in CRAO, ultimately causing retinal ischemia and vision loss [4]. Morbidity is high, with over 80% of patients having an initial visual acuity (VA) of count vision fingers or worse [5], and less than 20% of patients will recover a functional VA [6]. Interestingly, up to 25% of CRAO patients will have a spared cilioretinal artery and may present with relatively preserved VA [7].

The physical examination will reveal a patient with monocular vision loss, impaired color vision, and relative afferent pupillary defect [5]. Retinal edema, a cherry red spot, attenuation of retinal arteries, segmentation of retinal artery blood columns, and occasionally retinal artery emboli may be visualized on fundoscopic exams [7]. Systemic disease processes are associated with the development of CRAO, including hyperlipidemia; carotid artery disease; cardiac disease, including ischemic and structural heart disease; diabetes; vasculitides, including giant cell arteritis (GCA); thrombophilias; hemoglobinopathies, including sickle cell disease; orbital trauma or surgical procedures; renal disease and hemodialysis; smoking; oral contraceptives; and cocaine use, among others [8-10].

Treatment of CRAO is heterogeneous among academic centers. It includes interventions such as vasodilator therapy, orbital massage, laser embolysis or embolectomy, reduction of intraocular pressure (mechanically via anterior chamber paracentesis or trabeculectomy, or medically via intravenous acetazolamide and mannitol, or topical beta-blockers), hyperbaric oxygen therapy, intra-arterial thrombolysis, or intravenous thrombolysis (IVT) [11].

The use of IVT for CRAO has been studied since the 1960s [5]. However, data show only 5.8% of CRAO patients receive IVT [12]. Given this disease's acute and sight-threatening nature, emergency physicians (EPs) will inevitably evaluate and manage CRAO patients throughout their careers. When caring for patients with CRAO, EPs may be overwhelmed with available treatment options. While there are no guideline-endorsed treatments for CRAO, and despite the lack of randomized controlled trials (RCTs), IVT has emerged as a potential therapeutic for this condition [5], which has posed some controversy [13]. We will explore the most current literature on IVT as a treatment for CRAO and provide EPs with a focused, up-to-date, evidence-based background on this topic. 

## Review

IVT is the standard of care for acute ischemic strokes presenting within less than 4.5 hours following symptom onset [14]. CRAO is a subtype of ischemic stroke affecting the retina, which can cause sudden, permanent vision loss if not treated promptly. No guideline recommendations exist for IVT in CRAO; however, literature exists on the topic. One of the earliest, a 2002 study by Kattah et al., described their initial experience using intravenous recombinant tissue-type plasminogen activator (tPA) in 12 patients presenting with CRAO within 24 hours. In that initial pilot study, 10 of the 12 patients had VA improvements, suggesting the potential role of IVT in treating CRAO [15]. This narrative review will focus on the most current literature on the topic and highlight the benefits and risks of IVT in CRAO.

Methods

The author searched PubMed for articles containing the keywords “central retinal artery occlusion” AND “thrombolysis,” “thrombolytic,” “alteplase,” or “tissue plasminogen activator.” To select the most current literature, the PubMed database was screened for articles from 2020 to June 30, 2023, and filtered for full-text, English, systematic reviews, reviews, meta-analyses, and clinical trials, yielding a total of 3,972 articles. Individual case reports were excluded from the primary search. Upon review, 3,959 were excluded for nonrelevance as determined by the author, redundancy, or duplication. Nonrelevance was determined through a screening process of titles and abstracts. This screening process excluded papers that did not have a topic-specific focus on IVT as a treatment for CRAO, and omitted papers concentrating only on intra-arterial thrombolysis. Ultimately, 13 recent papers met these inclusion criteria. A narrative review of these papers follows. 

Although great care was taken to include up-to-date and relevant studies on this topic, selection bias may still exist, as this is a narrative review researched and written by a single author.

Literature overview

In 2020, Grory et al. published a review on IVT in CRAO [16], which evaluated four studies [17-20]. The first, a patient-level meta-analysis by Schrag et al., included nine studies and 147 patients [20]. Thrombolytic therapy varied between studies, two using alteplase, four using streptokinase, and three using urokinase. Thirty-four patients received IVT within 4.5 hours of symptom onset. Of those 34 patients, 17 (50%) had recovery of functional VA. This review also evaluated two prospective interventional case series by Nedelmann et al. and Schultheiss et al. and one retrospective analysis by Préterre et al., with recovery of VA in 36.3%, 30%, and 55.2% of patients, respectively [17-19]. From these studies, only one patient experienced symptomatic intracerebral hemorrhage (ICH); however, in this case, heparin was started immediately following IVT, which constituted an American Heart Association (AHA) guideline protocol violation [16].

In the same year, Grory et al. also published a cohort study that enrolled 112 patients presenting within 48 hours of symptom onset with a VA of <20/200 [21]. Of the cohort, 25 patients were treated with IVT, and 44% had recovery of VA [21]. Side effects were rare, with one patient developing an asymptomatic ICH. An updated patient-level meta-analysis was also included, encompassing 238 patients. Of those patients, 67 received IVT within 4.5 hours, with a recovery rate of 37.3% [21].

Another 2020 paper by Dumitrascu et al. evaluated seven papers from 1995 to 2018, encompassing 111 CRAO patients who received IVT [22]. Three of the papers evaluated were also included in the review by Grory et al. Of the 111 patients, 60 (54%) received IVT within 4.5 hours [22]. Standard alteplase dosing (0.9 mg/kg IV, maximum of 90 mg, with 10% bolus over 1 minute, followed by the remaining dose over 1 hour) was used in most studies; however, two case reports by Mames et al. [23] used an unspecified alteplase dosing and a case series by Hattenbach et al. [24] used 50 mg intravenous bolus over 60 minutes [22]. VA improvement following IVT varied between studies, with most showing benefit following very early IVT administration and one study showing overall visual improvement in 10 out of 12 (83.3%) of its subjects [22]. While several of the studies reported rare side effects, including four patients with ICH, one patient with vitreous hemorrhage, one patient with hematuria, one patient with angioedema, and one patient with bleeding from an abdominal aortic aneurysm, none of the patients who received IVT within 4.5 hours developed symptomatic ICH or ocular hemorrhage [22]. The article concluded that “CRAO patients should theoretically receive the same thrombolytic therapies, in the same time window, as patients with acute cerebral ischemia.”

In a 2021 systematic review conducted by Wang et al. [25], similar coverage was provided for the seven studies evaluated by Dumitrascu et al. Additionally, Wang et al. evaluated a study by Wu et al. [26], which examined the clinical efficacy and safety of combined tPA and anisodine therapy. Forty-eight subjects were enrolled and divided equally into control and treatment groups [26]. Of the 24 patients within the treatment arm, 22 cases (91.67%) demonstrated clinical efficacy in comparison to the control arm (17 cases, 70.83%) [26]. Twelve patients with CRAO who presented within 24 hours of symptom onset received tPA; 10 of the 12 (83.3%) had improvement in VA [26]. 

Schönecker et al. published a pilot study in 2022 investigating the efficacy and safety of IVT in patients with retinal infarction [27]. They enrolled 38 patients, 19 with CRAO, six with branch retinal artery occlusion (BRAO), and 13 with transient vision loss (TVL). Of the 19 patients with CRAO, nine were treated with IVT [27]. All other patients received standard-of-care treatment. Results demonstrated that the IVT group had a more significant difference between the modified Rankin scale at admission and discharge than the standard-of-care groups [27]. A single patient (11.1%) in the IVT group developed an asymptomatic ICH [27].

Finally, a 2023 paper by Raber et al. presented a retrospective observational study of CRAO patients treated either with IVT within 4.5 hours or conservative therapy [28]. They enrolled 37 patients, 16 in the IVT group and 21 in the conservative group [28]. Standard stroke-protocol alteplase dosing was given to IVT group patients [28]. Additionally, patients received so-called *minimal* alternative therapies, including bulbar massage (IVT group 29% vs. conservative group 57%) and intraocular pressure lowering medications (IVT group 40% vs. conservative group 70%), when directed by an ophthalmologist [28]. Of the 16 patients in the IVT group, 3 (19%) showed improvement in VA: one patient improved from blindness to severe visual impairment, and two patients improved from blindness to the ability to read and had better visual capabilities [28]. In comparison, none of the patients in the conservative group had improvement in VA [28].

What we can definitively conclude from these studies is unclear. Limitations include lack of RCTs, low-sample sizes, and heterogeneity of study designs, treatment arms, and outcome measurements. Several review papers have explored this available literature and attempted to provide direction for physicians treating CRAO patients; however, their endorsements for IVT vary.

A 2021 statement from the AHA concluded, “The current literature suggests that treatment with intravenous tissue plasminogen activator may be effective,” with the caveat that “the literature…is constrained by multiple variables” [5]. In a paper titled “Central Retinal Artery Occlusion: Can We Effectively Manage This Ocular Emergency in a Hospital Setting?” Jayasinghe et al. suggested, “Current data mandates that CRAO should be treated with the same urgency as an acute ischemic cerebral stroke”; however, regarding IVT states, “Treatments other than intra-arterial and intravenous thrombolysis have shown some improvement in the management of CRAO when provided as combination therapy” and “...it is crucial to conduct additional clinical trials on CRAO patients to identify the optimal combination of drugs required for optimal results in CRAO” [29]. A review by Madike et al. recommended, “If the patient's vision loss has been less than 4.5 h, IV tPA should be considered in collaboration with the stroke team. If the patient has had symptoms for over 4.5 h, the patient should be commenced on antiplatelet therapy and reviewed by the acute stroke unit for workup and secondary prevention” [30]. Yet another review by Janská et al. concluded, “It seems that the IVT efficacy and safety can be confirmed” [31]. Okonkwo et al. made a case for neuroprotective strategies in patients with CRAO, and on the subject of IVT state “...intravenous (IV) thrombolysis (IV-tPA) in CRAO though controversial, could hold some promise for a category of patients who can have treatment early and perhaps suffer from incomplete arterial occlusion” [32]. A 2023 Cochrane review, which looked at six RCTs, totaling 223 participants with acute nonarteritic CRAO, could not provide a strong recommendation for IVT in CRAO [11]. They cited study heterogeneity, with no study comparing the same interventions. They concluded, “Based on low‐certainty evidence, we found no significant difference in VA when tissue plasminogen activator (t‐PA) or transcorneal electrical stimulation (TES) was compared with placebo or existing treatments such as eyeball massage, oxygen inhalation, tube expansion, and anticoagulation” [11].

Virtually, all of these papers highlighted the need for further research, a focus on RCTs, larger sample sizes, standardized study designs, and measurement of clinically significant outcomes. Currently, three ongoing clinical trials aim to bridge this gap. THEIA (A Phase III Randomized, Blind, Double Dummy, Multicenter Study Assessing the Efficacy and Safety of IV THrombolysis (Alteplase) in Patients With acutE Central retInal Artery Occlusion) has an estimated enrollment of 70 patients with an estimated completion date of October 2024 [33]. Its primary outcome measurement is “visual acuity (VA) improvement after treatment” with a time frame of one month [33]. REVISION (Early Reperfusion Therapy With Intravenous Alteplase for Recovery of VISION in Acute Central Retinal Artery Occlusion) has an estimated enrollment of 1,400 patients with an estimated completion date of December 31, 2025 [34]. Its primary outcome measurement is “Functional recovery at visit 3” with a time frame of 30 days [34]. TenCRAOS (TENecteplase in Central Retinal Artery Occlusion Study) has an estimated enrollment of 78 patients with an estimated completion date of May 31, 2024 [35]. Its primary outcome measurement is the “proportion of patients with ≤ 0.7 logMAR visual acuity in the affected eye at 30 (±5) days after treatment, representing an improvement in visual acuity of at least 0.3 logMAR (intention-to-treat (ITT) analysis),” with a time frame of one month [35].

Where does this leave EPs? Again, there are currently no guideline-recommended treatments for reversing vision loss in patients affected by CRAO. The last practice guideline from the American Academy of Ophthalmology (AAO) states, “In general, there are no proven therapies or treatments for symptomatic artery occlusions” and “More aggressive treatments, such as thrombolysis…have associated risks and cannot be currently recommended in the absence of strong evidence-based data” [36]. In response, Grory et al. argued that “thrombolysis for acute CRAO is bolstered by 2 key observations,” the first being a “favorable safety profile” and the second recognizing that the “recanalization rate in response to tissue plasminogen activator is inversely proportional to the diameter of the implicated vessel in cerebral stroke” [37]. Although, until the completion of current and future RCTs, it is difficult to provide a robust and evidence-based recommendation for the use of IVT in CRAO.

The AHA and AAO, however, do give the general advice that patients with symptomatic CRAO should undergo emergent evaluation at the nearest stroke center, a fundoscopic exam be performed to rule out other causes of acute, painless vision loss (such as retinal or vitreous hemorrhage), that the patient be screened for GCA, and that an etiologic workup be performed urgently (e.g., evaluation for carotid artery disease, hypercoagulable states, atrial fibrillation, structural heart disease, etc.) [5,36]. Ultimately, the AHA urges, "We must develop systems of care for the urgent recognition, triage, and management of CRAO in a manner similar to cerebral ischemic stroke” [5]. And yet, one multicenter study showed that out of 9,511 telestroke encounters, only 49 (0.51%) were conducted for an acute eye complaint [38]. Furthermore, while five cases were categorized as having possible CRAO, and four of those patients presented within 4.5 hours, none received IVT [38]. The authors of this study ultimately concluded that evaluations for CRAO were less common than the disease prevalence and that overall, “current assessment of acute visual loss through telestroke is suboptimal” [38].

Current physician response to CRAO is affected by logistical factors, including comfort in making the diagnosis. EP management will likely be system dependent. EPs working in large academic institutions may find no difficulty arranging prompt consultations by stroke teams and in-house ophthalmologists. However, EPs practicing in rural or community hospitals without stroke teams or ophthalmology may find meeting AHA and AAO expert recommendations more challenging. Compound this with the fact that in one survey of EPs, only 28.9% felt comfortable diagnosing CRAO [39]. Without solid evidence or in-house support, they must rely on hospital-specific or local practice procedures or guidance from and transfer to tertiary care centers, which may delay time-sensitive treatments.

## Conclusions

At present, there is a deficiency of strong evidence for IVT for CRAO. However, results from previous studies point toward the potential use of IVT for patients affected by acute, symptomatic CRAO. While specific guidelines remain dim, current and future RCTs will help elucidate the possible benefits and risks of IVT for CRAO and shine a guiding light for EPs looking for efficacious treatment options for their patients. 
